# Saponins of *Trifolium* spp. Aerial Parts as Modulators of *Candida Albicans* Virulence Attributes

**DOI:** 10.3390/molecules190710601

**Published:** 2014-07-21

**Authors:** Aleksandra Budzyńska, Beata Sadowska, Marzena Więckowska-Szakiel, Bartłomiej Micota, Anna Stochmal, Dariusz Jędrejek, Łukasz Pecio, Barbara Różalska

**Affiliations:** 1 Department of Infectious Biology, Institute of Microbiology, Biotechnology and Immunology, University of Lodz, Lodz 90-237, Banacha 12/16, Poland; E-Mails: albudzal@biol.uni.lodz.pl (A.B.); bsad@biol.uni.lodz.pl (B.S.); mwiec@biol.uni.lodz.pl (M.W.-S.); bmic@biol.uni.lodz.pl (B.M.); 2 Department of Biochemistry, Institute of Soil Science and Plant Cultivation, State Research Institute, Pulawy 24-100, Czartoryskich 8, Poland; E-Mails: asf@iung.pulawy.pl (A.S.); djedrejek@iung.pulawy.pl (D.J.); lpecio@iung.pulawy.pl (Ł.P.)

**Keywords:** saponins, *Trifolium* spp, *Candida albicans*, virulence factors

## Abstract

The aim was to provide the insight into the biology of *C. albicans* influenced by undescribed yet properties of saponin-rich (80%–98%) fractions (SAPFs), isolated from extracts of *Trifolium alexandrinum*, *T. incarnatum*, *T. resupinatum* var. *resupinatum* aerial parts. Their concentrations below 0.5 mg/mL were arbitrarily considered as subMICs for *C. albicans* ATCC 10231 and were further used. SAPFs affected yeast enzymatic activity, lowered tolerance to the oxidative stress, to the osmotic stress and to the action of the cell wall disrupting agent. In their presence, germ tubes formation was significantly and irreversibly inhibited, as well as *Candida* invasive capacity. The evaluation of SAPFs interactions with anti-mycotics showed synergistic activity, mainly with azoles. Fluconazole MIC was lowered—susceptible *C. albicans* ATCC 10231 was more susceptible, and resistant *C. glabrata* (clinical strain) become more susceptible (eightfold). Moreover, the tested samples showed no hemolytic activity and at the concentrations up to 0.5 mg/mL did not reduce viability of fibroblasts L929. This study provided the original evidence that SAPFs of *Trifolium* spp. aerial part exhibit significant antimicrobial activity, by reduce the expression/quantity of important *Candida* virulence factors and have good potential for the development of novel antifungal products supporting classic drugs.

## 1. Introduction

The modern approach on the use of plant secondary metabolites for combating human and animal pathogens involve clarifying the mechanisms and targets of their activity, forming the basis for more effective and safe use. Besides bacteria and viruses, dermatophytes, dimorphic fungi (mainly *Candida* spp.) and some species of molds are a cause of big health problems. *C. albicans* is a yeast that resides as commensals in the oral cavity and the gastrointestinal tract, however, incidence of symptomatic infections increases significantly. Therefore, knowledge of the mechanisms of pathogenicity used by *C. albicans* during infection is crucial for the development of new antifungal therapies, diagnostics and prophylaxis [1,2,3]. In this study, we asked the questions whether and how we can change some significant phenotypic characteristics of *C. albicans*. We report results of experiments providing the insight into the biology of *C. albicans* influenced by the action of saponin fractions prepared from the aerial part extracts of selected species of *Trifolium* L. genus—one of the largest genera in the *Fabaceae* family. Clovers are used mainly as a fodder and pasture crops but they also gain interest due to the content of secondary metabolites, in particular saponins and flavonoids. They are popular food additives or diet supplements and also find application in pharmaceutical or cosmetic industries [4]. The production of saponins by plants is an important part of their defense against pathogens and herbivores; however, they are well-known to possess a much broader spectrum of properties, such as hemolytic, anti-inflammatory, cytotoxic, and antitumor activity [5]. The most attractive issue in our research topic is the antimicrobial activity of saponins. We believe that their new application, beyond the above mentioned industrial sectors, is possible. New antimicrobials could be derived from these natural easily available phytocompounds, for supplementing and/or supporting classic pharmacological agents [6]. Specifically targeted virulence factors have been proposed as a new and promising approach in the search for new therapeutic options [3,7,8]. These approaches are described in the present article.

The plant species selected for this study were *T. alexandrinum* (berseem clover), *T. incarnatum* (crimson clover) and T*. resupinatum* var. *resupinatum* (Persian clover)—the most important members of the annual of *Trifolium* species. Previous studies had revealed that the amount, composition and biological activity of saponins in their seed extracts were different [9,10,11]. It is interesting to evaluate these parameters in relation to the aerial parts of these plants. As the main goal of the present study was to test the specific antifungal properties, in order to verify our hypothesis that saponins activity seem to be very promising in the context of their possible medical applications.

## 2. Results and Discussion

Research into new treatment options effective in combating infections involves a search for substances with different types of activity. They may have not only direct antimicrobial activity or exhibit a synergistic effect with conventional pharmaceutics, but also reduce the expression of pathogen virulence factors or activate host defense mechanisms [3,7,8,12]. The saponin fractions (SAPFs), obtained for the first time from the aerial parts of *T. alexandrinum*, *T. incarnatum*, *T. resupinatum* var. *resupinatum*, fulfill most of the above expectations. Our studies have showed novel pharmacological properties of these saponins, documenting their influence on more than usually tested pathogenesis attributes relevant for *Candida* patomechanisms [11,13]. The analysis of *Trifolium* extract fractions by HPLC-ELSD revealed their total saponin content in relation to all presented peaks, and amounted to 79.92%, 97.83% and 90.21% of saponins for *T. alexandrinum*, *T. incarnatum*, and *T. resupinatum* var. *resupinatum*, respectively. The tentative identification of the major components of each fraction was carried out through a careful study of their ESI-MS/MS fragmentation pattern and comparison with the pre-existing literature data. Two triterpenoid glycosides, *i.e*., soyasaponin Bb (soyasaponin I) and soyasaponin βb (soyasaponin I conjugated at the 22-position with DDMP), previously characterized in the seeds of several clovers, were found as a major saponins in three tested species [14]. The SAPF of *T. alexandrinum* contained mainly these two compounds, which represented 85.95% and 5.91% of total saponins for soyasaponin Bb and βb, respectively. The chromatogram of crimson clover fraction also composed principally of two aforementioned triterpenoid glycosides, but in different amount ratio as compared to the previous fraction. Thus, the main saponins of the SAPF of *T. incarnatum* were soyasaponin βb (48.19% of total saponins) and soyasaponin Bb (43.46% of total saponins). The composition of saponin fraction of *T. resupinatum* var. *resupinatum* was the most complex among tested fractions, and consisted of several peaks from which major three were identified as soyasaponin Bb, Bb’(soyasaponin III) and βb [14]. Their quantitative ratio was as follows, 45.75%, 15.03% and 12.36% of total saponins for soyasaponin Bb, Bb’ and βb, respectively.

According to above analysis the saponin fraction of *T. alexandriunum* contained the residues of flavonoids (apigenin and its glucosides), that amounted to 15.20% of total peaks presented in the chromatogram. However, the final concentration of apigenin and derivatives in the sample used in our experiments was far too low to cause the observed biological effects. For example, in the publication by Cheah *et al.* [15], the minimal concentration of apigenin inhibiting growth of *C. albicans* was rated at 125 µg/mL, while in the saponin—rich fraction obtained from the extract *T. alexandrinum* its concentration could reach 8 times lower level (a maximum concentration of only about 18 µg/mL). Even taking into account the synergistic effect of the ingredients of plant extract, it is unlikely that such a concentration of apigenin could affect the final outcome of its antifungal action. This conviction is supported by data from the literature, as well as by own experience of the subject. Additionally, the experimental data on the viability and function of mammalian cells influenced by apigenin indicate that this flavonoid at a 25–40 µM concentration affected cell growth and was cytotoxic [16,17]. Thus, if present in our product apigenin (not saponins) showed activity, this should occur during the assessment of cytotoxicity of SAPF and it did not happen. According to the results of an MTT- reduction test with L929 fibroblasts, none of the saponin fractions, used at the concentrations range of 0.003–0.5 mg/mL, did not reduce vital cell numbers (*p* > 0.05) after 0.5 h of incubation (data not shown). Values of IC_50_ after 24 h of co-incubation were as follows: for *T. alexandrinum*—0.125 mg/mL; for *T. resupinatum* and *T. incarnatum*—0.25 mg/mL. Moreover, these SAPFs showed no hemolytic activity on TSA/5% of human erythrocytes, while the diameter of the zones of hemolysis induced by standard saponin—Quil A (from *Quillaja saponaria*) were, respectively, 3.0 ± 0.67 mm for 0.25 mg/mL, 3.94 ± 0.5 mm for 0.5 mg/mL, and induced by Triton 1% X-100 (positive control)—9.33 ± 0.94 mm. Thus, the antifungal effects of saponins observed in further studies were caused by them, but not by additional components.

Minimal fungistatic concentrations of SAPFs against *C. albicans* ATCC 10231 and *C. glabrata* clinical isolate exceeded the concentration of 1 mg/mL (w/v). Based on this, the concentrations of 0.125, 0.25 and 0.5 mg/mL, which did not cause yeast growth inhibition, were arbitrarily considered as subMICs of the SAPFs and were further interchangeably used.

**Figure 1:**
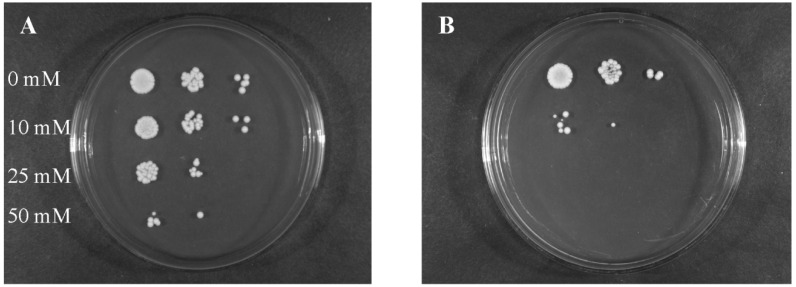
The oxidative stress tolerance assay of C. albicans ATCC 10231 pre-cultured for 24 h at 35 °C on control SDA (**A**) or on SDA + *T. alexandrinum* SAPF (0.5 mg/mL); (**B**) The inocula of yeasts prepared in this way, were further incubated with different concentrations of hydrogen peroxide for 1 h at room temperature, diluted (10^5^ to 10^3^ cells/mL) and spotted (5 μL) on YPG plates. Intensity of growth (micro- and macrocolony morphology) was evaluated after the following 48 h of incubation at 30 °C. Four independent experiments were performed. In Figure 1, the representative data are shown.

**Figure 2:**
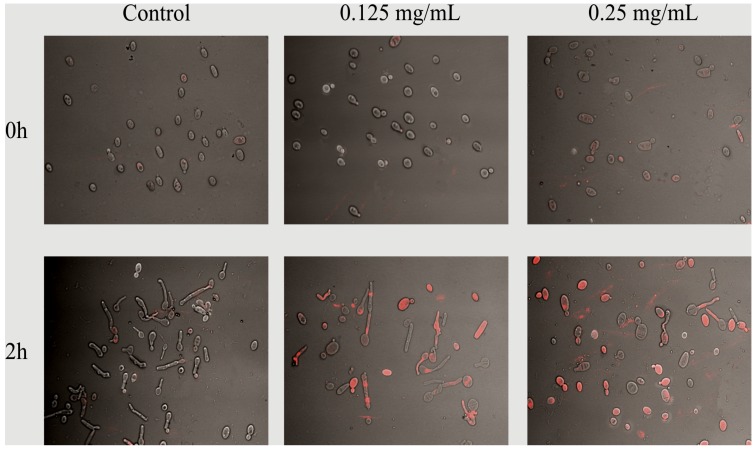
*C. albicans* control and *T. alexandrinum* SAPF—treated cells (at concentration of 0.125, 0.25 mg/mL) stained after 0 and 2 h of co-incubation with propidium iodide (60 µM). Undamaged cells (gray/transparent) and the cells with impaired cell wall permeability (red) were determined using a Confocal Laser Scanning Microscope (PI fluorescence detected at the excitation of 543 nm). The presented images are representative from two independent experiments.

The first question that we asked, is it efficient oxidative stress response of *Candida* will be affected by saponin action. Such a response may be of clinical interest, since it is important for *C. albicans* invasion and colonization of host tissues and survival within the host cells (phagocytes) in the course of an *in vivo* infection [18,19]. It is known that *C. albicans* strains show *in vitro* a high natural resistance to H_2_O_2_ and various protocols of treatment with this agent (concentration- and time-dependent) have distinct effects on antioxidant enzymes (catalase, superoxide dismutase, glutathione oxidase) expression [18]. In our study, *C. albicans* ATCC 10231 cells when exposed to SAPFs (0.5 mg/mL), exhibited lower oxidative stress tolerance after their treatment with various doses of hydrogen peroxide than the control cells. The most significant increase in susceptibility to oxidative stressor was caused by *T. alexandrinum—*derived saponins (Figure 1). Spot plating of the pre-exposed yeasts on media containing different concentrations of the cell wall damaging agent—Calcofluor White, or media containing NaCl as osmotic stress factor, resulted in a reduction of visible growth intensity and delay of the growth time. However, the last two effects were not statistically significant (data not shown). In this part of the study our results have shown that saponins may cause changes in the composition of the cell wall of *Candida*, since its sensitivity not only to hydrogen peroxide but also slightly to Calcofluor White, have increased. It has been reported that Calcofluor binds to β-linked fibrillar polymers, interferes with chitin assembly resulting in growth rate reduction, and alters incorporation of mannoproteins into cell wall. Therefore, it cannot be ruled out that the architecture of the cell wall proteome might be changed by possibly preventing correct positioning and anchoring of cell wall localized superoxide dismutase or other proteins that are directly or indirectly responsible for countering stress damage [20,21]. Indirect evidence for the change in cell wall permeability of *Candida* caused by the action of saponin was delivered in experiment, wherein the yeast after a 2 h pre-incubation were stained with propidium iodide, and observation of cell morphology was made under a microscope. The results are shown in Figure 2.

**Figure 3:**
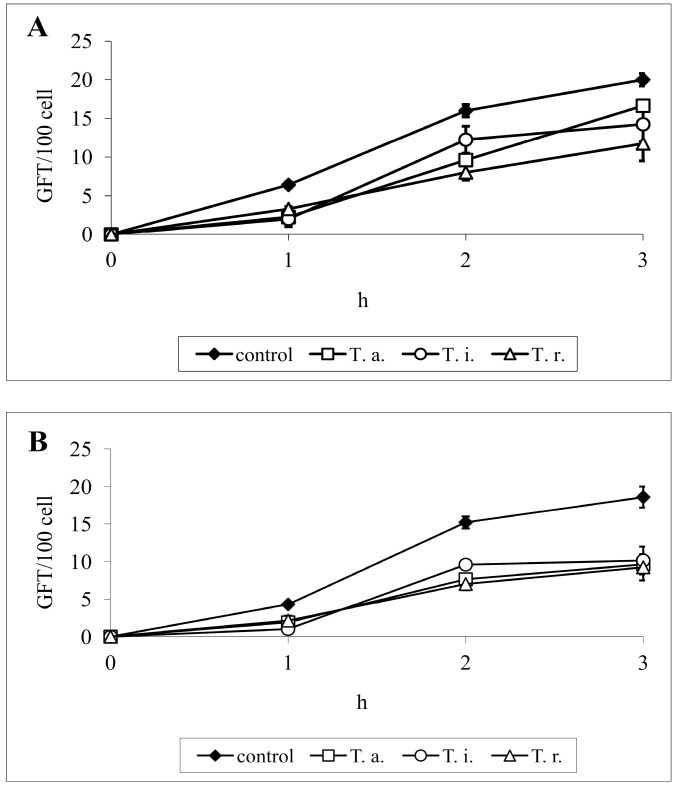
Kinetics of *C. albicans* ATCC 10231 germ tube formation after 1, 2, 3 h of incubation in RPMI-FCS medium in the presence of *T. alexandrinum* SAPF (**A**)—0.125 mg/mL or (**B**)—0.25 mg/mL. The results are expressed as mean GFT/100 cells ± S.D., evaluated at each time point. *T. a.—Trifolium alexandrinum*; *T. i.—T. incarnatum*; *T. r—T. resupinatum.*

Very important and well known virulence factors of *Candida* cells are hydrolytic enzymes such as proteases, lipases and phospholipases, playing a role in nutrition, adhesion to host cells, and tissue destruction. The most important are Sap (secreted aspartyl proteinases)—Sap 1–3 are secreted by blastospores, Sap 4–6 are released mainly by filamentous forms, and Sap 9 and 10 are strongly associated with the cell wall of both morphotypes. Protease activity is complemented by the action of phospholipases, which are enzymes responsible for the hydrolysis of one or more ester bonds in the cell membrane glycerophospholipids. Among the seven known types of *C. albicans* secreted phospholipases, the most important seems to be a phospholipase B, which causes the release of fatty acids from phospholipids and lysophospholipids, playing an important role in the penetration of host tissues [2,19,22]. Our second goal, justified by the above information, was to test enzymatic activity of yeasts pretreated with saponins. Using a semi-quantitative API ZYM test, it was demonstrated that *C. albicans* reference ATCC 10231 strain exhibited the activity of 9/19 hydrolytic enzymes, *i.e*., alkaline phosphatase, esterase (C4), esterase lipase (C8), leucine and valine arylamidase, acid phosphatase, naphthol-AS-BI-phosphohydrolase, α-glucosidase, and N-acetyl-β-glucosaminidase. Treatment of this strain with SAPFs (at 0.5 mg/mL) revealed a statistically significant decrease in the release of some of the enzymes, including acid and alkaline phosphatase, naphthyl-AS-BI-phosphohydrolase and *N*-acetyl-β-glucosaminidase. The production of other enzymes was also slightly affected (Table 1). The observation that enzymatic activity of *Candida* treated with SAPFs was significantly decreased is important in the light of the results of other experiments on the formation of filaments. In the constant presence of SAPFs at 0.25 mg/mL in RPMI-FCS medium, germ tubes (GT) formation by *C. albicans* ATCC 10231 strain was significantly inhibited (Figure 3B). The number of GT-positive cells per 100 cells, evaluated after 3 h of co-incubation, dropped from 18.6 ± 1.4 to app. 9.2 ± 1.75 − 10.9 ± 0.4. The mean percentage of GT reduction caused by the individual SAPF only slightly differed (42%–54%) indicating that all SAPFs were equally potent in this respect. Interestingly, the observed reduction in *C. albicans* ability to form filaments was irreversible, as verified during prolonged incubation of preincubated yeasts, for a total of 24–48 h. During this time *C. albicans* control cells formed aggregates (microcolonies) interspersed with filaments and true hyphae (mycelium) (Figure 3A,A_1_), while yeasts treated earlier with subMIC of SAPFs had a form of short blastospore-budding chains (Figure 3B,B_1_).

**Table 1:** API ZYM test for *C. albicans* ATCC 10231 enzymatic activity under the influence of *Trifolium* spp—derived SAPFs. The mode of phytocompounds action, used at subMICs (0.5 mg/mL) was as described in the Experimental section.

SAPFs	Hydrolytic Enzymes * Activity [nmol]								
	E2	E3	E4	E6	E7	E11	E12	E16	E18
Control (−)	*10*	*30*	*20*	*30*	*10*	*40*	*30*	*20*	*30*
*T. alexandrinum*	0	20	20	20	10	0	10	30	0
*T. incarnatum*	0	30	10	30	10	10	10	30	10
*T. resupinatum*	0	30	20	30	10	20	20	20	20

*: E2—alkaline phosphatase; E3—esterase (C-4); E4—esterase lipase (C-8); E6—leucine arylamidase; E7—valine arylamidase; E11—acid phosphatase; E12—naphthol-AS-BI-phosphohydrolase; E16—α-glucosidase; E18—N-acetyl-β-glucosaminidase.

**Figure 4:**
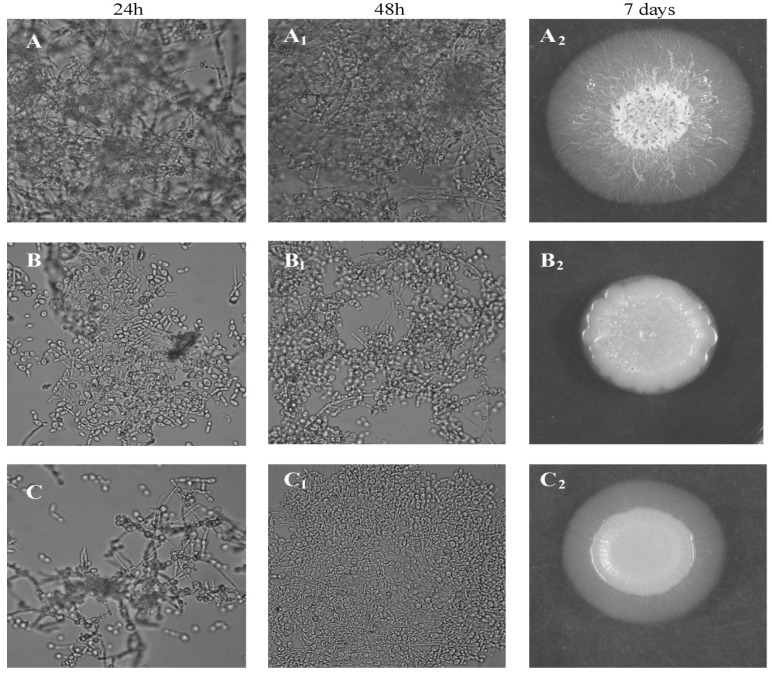
Hyphae formation (left and middle columns) by *C. albicans* ATCC 10231 strain cultured in RPMI-FCS medium in the absence (**A**, **A_1_**) or presence of *T. alexandrinum* SAPF at concentration of 0.25 mg/mL (**B**, **B_1_**) or at concentration of 0.125 mg/mL (**C**, **C_1_**), tested after 24 h or after 48 h. At the indicated time points of culture, samples were withdrawn, evaluated microscopically (light microscope 400× magnification). Mycelium formation (right column) by *C. albicans* ATCC 10231 colonies grown for 7 days at 30 °C on (**A_2_**)—control Spider medium; (**B_2_**)—Spider medium + SAPF 0.25 mg/mL; (**C_2_**)—Spider medium + SAPF 0.125 mg/mL. The presence or lack of hyphal growth at the colony edges was determined using a stereomicroscope (12× magnification). The presented images are representative from two independent experiments.

This effect was correlated with the reduction in the invasive capacity of the yeasts, as stated in the standard test simulating their penetration into tissues. Filaments (hyphal growth examined using a stereomicroscope), seen at the edges of *Candida* colonies grown in the control were large and formed an extensive spider branched zone around a dense mass of mycelium, while *C. albicans* spotted on the Spider agar containing individual SAPF (0.25 mg/mL) were unable to penetrate this medium. The effect of SAPFs used at a lower concentration (0.125 mg/mL) was weaker, however, also noticeable. An example result of *T. alexandrinum* SAPF activity is shown in Figure 4A_2_,B_2_,C_2_ (right column).

It is assumed that the most enzymatically active parts are apical tips of young hyphal cells which are best suited to adhere to and invade host tissues. Yeast treated earlier with subMIC of SAPFs took at the tested end-points a form of short blastospore-budding chains. It is an important achievement, since young buds might be more susceptible to antifungals and, *in vivo*, to the activity of host immune effector mechanisms. These results suggest that the morphological transformation of *C. albicans* cells under the influence of SAPFs is completely blocked. At this stage of the study, however, we do not know what the precise mechanism of their action is. According to Biswas *et al*. [20] and Thompson *et al*. [21] and others [2,3,19] filamentation is controlled by a very complex network of regulatory pathways that converge onto specific transcriptional regulators.

Anyway, the capacity of each compound to inhibit germ tube formation could be an important factor to assess its antifungal activity. The most important achievements involved reduced enzymatic activity of *Candida*, as increased sensitivity of their cells to oxidative stress, and impaired morphological transition, as well invasive properties. The yeast-to-hypha transformation has been shown to be one of the most important among several virulence attributes that enable *C. albicans* to invade human tissues.

Prepared saponins had no direct strong antifungal activity within the concentration range tested. However, when used at subMIC together with anti-mycotic drugs, they exhibited significant synergistic activity, mainly with drugs from the azole therapeutic group (Table 2). The results from a disk-diffusion assay show that SAPFs (mixed with SDA medium), combined with azoles (miconazole, clotrimazole, ketoconazole, econazole containing disks) caused prominent effect. In contrast, amphothericin B, nystatin, natamycin and flucytosin combined with SAPFs had no, weak or insignificant synergistic mode of interactions.

**Table 2:** Synergistic activity of *Trifolium* spp—derived SAPFs at subMIC with selected anti-mycotic chemotherapeutics. AmB—amphotericin B; KCA—ketoconazole; ECN—econazole; NY—nystatin; FY—flucytosin.

SAPFs	Anti-Mycotic Agent, Growth Inhibition Zone [mm ± S.D.]						
	AmB ^x^	KCA ^x^	KCA ^xx^	ECN ^x^	ECN ^xx^	NY ^x^	FY ^x^
Control (−)	12.0 ± 0	0	20.0 ± 0.0	11.0 ± 0.0	20.5 ± 0.5	18.0 ± 2.3	0
*T. alexandrinum*	11.0 ± 0	17.0 ± 0.0	-	14.0 ± 0.0	-	19.5 ± 0.7	0
*T. incarnatum*	11.0 ± 0.5	18.5 ± 0.7	-	14.5 ± 0.0	-	18.0 ± 0.0	0
*T. resupinatum*	10.5 ± 0.5	16.2 ± 0.9	-	12.0 ± 0.0	-	18.0 ± 0.5	0

*C. albicans* ATCC 10231 was spread on control SDA or SDA + SAPFs (0.25 mg/mL). The disk-diffusion (Mastring-S) test was performed as described in the Material and Methods section and the growth inhibition zones (mm **±** S.D.) were measured after incubation at 37 °C for 24–48 h. ^x^—a zone of complete growth inhibition; ^xx^—a zone with microcolonies.

The interesting effect of saponins in combination with some azoles is worth mentioning. The large zone of microcolonies around the disk with ketoconazole observed in the control (20 mm) disappeared under the influence of SAPFs subMIC, while previously absent zone of complete growth inhibition occurred. Its diameter was from 14.2 mm to 20.5 mm, depending on the SAPFs type. A similar effect was observed around the disk with econazole (Table 2).

**Figure 5:**
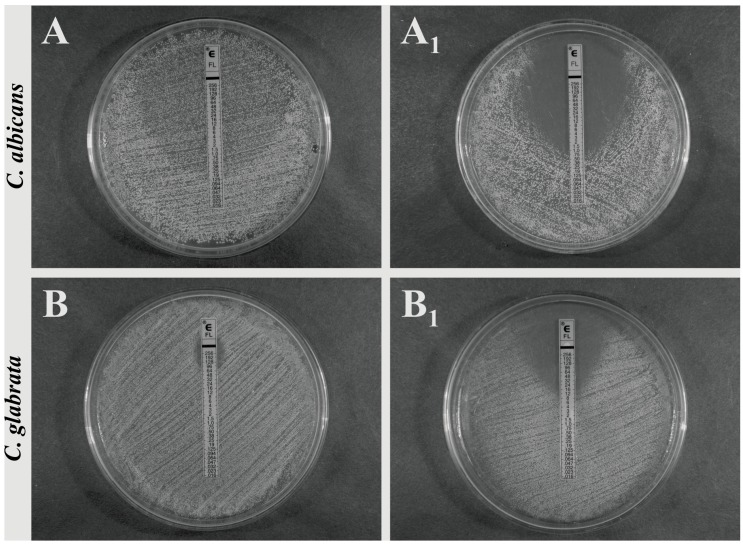
Synergistic activity of *T. alexandrinum* SAPF (0.25 mg/mL) with fluconazole towards *C. albicans* ATCC 10231 and *C. glabrata* clinical strain, measured by the antibiotic gradient strip (E-test). The growth inhibition zones/MIC were measured after yeasts growth at 37 °C for 24–48 h on control medium: RPMI-1640 with 2% glucose (**A**,**B**) or on medium + SAPF (**A_1_**,**B_1_**). The test end-points were evaluated according to the manufacturer’s recommendations. The presented images are representative from two independent experiments.

Since the disc diffusion method is only a semi-quantitative test, the extension of the study involved the evaluation of MIC value changing upon the SAPFs influence, using a strips of ellipsometric test (Etest), containing a representative triazole (fluconazole, FLC). Its MIC against *C. albicans* ATCC 10231, measured according to the CLSI recommendation (80% growth inhibition), was 0.25 mg/L. In the case of yeasts treated with individual SAPFs, the end-point value as such did not change, while the zone with a lawn of microcolonies within a discernable ellipse disappeared and complete growth inhibition (100%) was observed (sharp end point of 0.75 mg/L could be noticed). The effect of *T. alexandrinum* SAPF which was the most significant, is presented in Figure 5A,A_1_.

Most interestingly, when fluconazole-resistant *C. glabrata* clinical strain (MIC FLC > 64 mg/L) was used for the same purpose, the effect of synergism was also seen. FLC MIC of *C. glabrata* strain was decreased eightfold by the action of subMIC of SAPFs, from 64.0 mg/L to the level of 8.0 mg/L. Such evident effect was only observed while using *T. alexandrinum* saponins (Figure 5B,B_1_). In other cases, the effect was noticeable but clearly weaker (data not shown).

Why are saponins so interesting in this respect? Firstly, these products are characterized by wide antimicrobial activity. Secondly, saponins differ in their chemical structure and characteristics showing also antioxidant, anti-inflammatory, and anti-apoptotic properties. In order to combat infection, their hydrophobic constituents contact directly the phospholipid bilayer of the microbial cell membrane, leading to an increase in the ion permeability, leakage of vital intracellular constituents or impairment of the pathogen enzyme systems and their respiration, as well as inhibition of protein synthesis and assembly. Their antifungal properties are also related to the ability of the main constituents to pass through the thick fungal cell wall and settle between fatty acid chains of lipid bilayers, disrupting lipid packaging and altering the structure of the cell membrane [4,5].

The latter property suggests that saponins can be used to support the activity of antifungal drugs targeting sterol compounds of the cytoplasmic membrane (polyenes, azoles). In the present study we have proved such a possibility. An interesting result of SAPFs combination with ketoconazole and econazole was shown. Their fungistatic action was replaced by a complete growth inhibition due to SAPFs presence in the medium. It is also a point of interest that synergistic interactions of SAPFs with representative triazole—fluconazole (FLC) against *Candida* strains with different susceptibility was obtained. Using a quantitative E-test, it was observed that subMIC of SAPFs made susceptible *C. albicans* more susceptible, and what is even more important, the resistance of *C. glabrata* strain decreased.

Our observation is that the level of resistance to fluconazole can be decreased by the action of SAPFs subMIC encourages further research in this area. Recently, fluconazole-resistant *C. albicans* strains and intrinsically resistant *Candida* species such as *C. glabrata* have been emerging in immunocompromised patients treated with FLC for therapy or prophylaxis. Trying to explain the mechanism of synergistic action of saponins with fluconazole, we have to go back to the determinants of fungal resistance to this drug. The molecular basis of fluconazole resistance includes modifications in the *ERG11* gene encoding the main enzymes of ergosterol biosynthesis, overexpression of genes encoding efflux pumps, and others which have not been well defined yet [23,24]. Based on the results of the performed experiments, we cannot identify the exact mechanism responsible for the synergism of fluconazole and saponins. However, it can be tentatively suggested that it is due to the facilitated penetration of the drug into the cell, which is high enough that the membrane transporters are becoming less effective. This suggestion can be confirmed by the results indicating the absence of clear synergism between saponins and polyene anti-mycotics such as amphotericin B and nystatin. It is known that they are not substrates for efflux pumps [25].

Considerations as to which of the examined clover species is the best source of saponins having the desired properties should be related to an analysis of their concentration, composition and, first of all, of course their impact on the tested attributes of *Candida*. Oleszek and Stochmal [9] studied the occurrence and concentration of soyasapogenol B glycosides in the seeds of 57 clover species, including *T. alexandrinum*, *T. incarnatum*, *T. pratense*, *T. resupinatum* var. *resupinatum* and *T. resupinatum* var. *majus* Boiss. In the case of berseem clover, soyasaponin I and its 22-*O*-diglucoside were quantified, and astragaloside VIII was also detected but in a very low amount, while soyasaponin Bb and its 22-*O*-diglucoside were found in the crimson clover (*T. incarnatum*). Similarly, soyasaponin I was the most abundant saponin in the Persian clover (*T. resupinatum*) varieties.

It appears that saponin fraction of *T. alexandrinum* (berseem clover), containing mainly soyasaponin Bb (85.95% of total saponins) and accompanying soyasaponin βb (5.91% of total saponins), cause the most significant effects. The SAPFs of *T. incarnatum* and *T. resupinatum* var. *resupinatum* characterized by either similar saponin profile with different ratio of major compounds or different saponin profile, in relation to the previous one, were less active. Numerous studies have revealed that the distribution and composition of saponins in different organs and tissues of plants are dependent on the stage of ontogenesis and variable environmental factors [9,11,26]. It is so important that the only standardized plant preparations, with a well-documented biological activity, have a chance to exist as a safe medical products, supporting the currently used therapeutics (a checkerboard assay is needed). Ensuring that objective is also justified by the new approach to combating infectious diseases, which is the use of immunomodulatory activity of phytochemicals.

## 3. Experimental Section

### 3.1. Plant Material and Fractionation

Seeds of authenticated *Trifolium alexandrinum*, *T. incarnatum*, *T. resupinatum* L. var. *resupinatum*, were obtained from Genebank, Zentralinstitute für Pflanzen-genetik und Kulturpflanzenforschung (Gatersleben, Germany). Seeds were planted in an experimental field of the Institute of Soil Science and Plant Cultivation in Pulawy (Poland). The aerial parts of the plants were harvested at the beginning of flowering, lyophilized and finely powdered. Then, samples were defatted with CHCl_3_ (Soxhlet apparatus) and extracted twice with 80% MeOH. The extracts were concentrated, the residues were re-suspended in water and loaded onto a LiChroprep RP18 (40–63 μm, 60 × 100 mm, Merck, Germany) glass column. The column was washed with water and then with 40% MeOH to remove sugars and phenolics. Saponins were eluted with 85% MeOH—solvent evaporation followed by freeze-drying yielded crude saponin fractions [27].

### 3.2. Novel Chromatographic and Mass Spectrometric Analysis of Fractions

Redissolved fractions were subjected to chromatographic and mass spectrometric analysis to evaluate their saponin profiles and total saponin content. A Surveyor HPLC system equipped with a PAD detector and coupled to an LCQ Advantage Max (Thermo Fisher Scientific, Waltham, MA, USA) ion trap mass spectrometer (MS) were used. A reverse phase Waters Xbridge BEH C18 column (2.5 μm, 150 × 3 mm) was applied for this purpose. Samples were separated using a linear 50 min gradient from 20% to 50% MeCN in 0.1% formic acid with 0.3 mL/min flow, and the column temperature was held at 50 °C. The chromatograms were examined with a PAD detector set at 210 nm. The MS was operated in the negative electrospray (ESI^−^) mode with the following ion source parameters: spray voltage 3.9 kV, capillary voltage −47 V, tube lens offset −60 V, capillary temperature 250 °C. Full scan spectra were acquired in the *m/z* range 150–2000. Automated MS/MS function was performed at 35% normalized collision energy by molecular ion isolation with a width of *m/z* 1.0 and maximum acquiring time of 250 msc. Data acquisition was conducted using the Xcalibur data system (version 1.3 SR1, Thermo Fisher Scientific, Waltham, MA, USA). The total saponin content in these fractions was measured using a Gilson’s GX-281 Series of HPLC System equipped with an ELS detector (_PREP_ELS™ II Detector), (Gilson, Middleton, WI, USA). Fractions were separated using a reverse phase column and chromatographic parameters that were identical to the aforementioned. The ELS detector was operated with the temperature of Spray Chamber (SC) and Drift Tube (DT) at 35 °C and 65 °C, respectively. Peaks corresponding to saponins were integrated and expressed as an appropriate percentage of all peaks presented in the chromatograms.

### 3.3. Hemolytic and Cytotoxic Activity of Saponins

Hemolytic activity of fractions was tested on tryptic-soya agar (BTL, Poland) containing 5% of human erythrocytes (TSA + E). Five microliters of the samples, at the concentrations of 0.125, 0.25 and 0.5 mg/mL, was applied to TSA + E, yielding a circular inoculation site (about 5 mm in diameter) and then incubated at 37 °C for 24 h. A transparent zone around the spots was considered as positive hemolytic activity. Purified saponin from *Quillaja saponaria*—Quil A (Superfos Specialty Chemicals, Denmark), prepared in the same concentration range, was used as a standard. Triton X-100 (1% v/v in PBS; Sigma, Saint Louis, MO, USA) was used as a positive control.

The L929 cells (ATCC cell line CCL 1, NCTC clone 929) at the density of 1 × 10^6^ cells/mL in RPMI-1640 medium with l-glutamine, NaHCO_3_ (Sigma, Saint Louis, MO, USA), 1% (v/v) penicillin/streptomycin (Sigma, USA), and 10% (v/v) heat inactivated fetal bovine serum - FBS (Cytogen, Poland) was seeded into a 96-well tissue culture plate (Nunc, Denmark) for 24 h at 37 °C, 5% CO_2_. Then, the culture medium was replaced with 200 μL of the medium containing the SAPFs in the concentrations range of 0.003–0.5 mg/mL (8 two-fold dilutions) and exposed for 0.5 h or 24 h. Control wells contained: Cells with culture medium (growth positive control), cells with 1.25% of ethanol (solvent control), the SAPFs alone (a negative control for the samples, Ks), and medium alone (a negative control for the cells growth, Km). The cytotoxicity of the SAPFs was evaluated by the 3-(4,5-dimethylthiazole-2-yl)-2,5-diphenyltetrazolium bromide (MTT)—reduction assay as described earlier [28]. The absorbance (A) was read at a wavelength of 550 nm using a microplate reader (Victor2, Wallac, Turku, Finland). The percentage of cell viability was calculated as follows: cell viability (%) = [(A_550_sample − A_550_Ks)/(A_550_ growth positive control − A_550_Km) × 100]. A dose-response curve was derived from 8 concentrations in the test range using 4 wells per concentration to determine the mean of each point.

### 3.4. Fungi

*Candida albicans* ATCC 10231 and *C. glabrata* clinical isolate were stored as stock cultures in 10% glycerol at −70 °C. Test suspensions were prepared freshly from the cultures grown on Sabouraud Dextrose agar (SDA, Difco, Laurence, KS, USA) for 24–48 h at 35 °C.

*Evaluation of the subinhibitory concentration (subMIC) of SAPFs and pre-treatment of the yeast.* The subMICs of SAPFs were determined using the agar dilution assay, with the final saponin concentrations: 0.125, 0.25, 0.5, 1.0 and 2.0 mg/mL. The end-point of the test system was defined after spreading *C. albicans* or *C. glabrata* suspensions on the surface of (a) the SAPFs-modified or (b) control SDA plates, and incubating them for 24–48 h at 35 °C. The highest concentration of each SAPF resulting in the lack of yeast growth inhibition was arbitrarily considered as subMIC.

### 3.5. Enzymatic Profile of C. Albicans

Activity of enzymes was measured by API ZYM (bioMerieux, Marcy-l’Etoile, France) strip test containing substrates for the detection of 19 hydrolases. The suspensions of *C. albicans* ATCC 10231 cells were prepared from the yeast cultured for 24 h, 35 °C on (a) SDA containing SAPFs at the final subMIC or (b) control SDA, and inoculated in the cupules of the test. Enzymatic activity was determined in nanomoles of the hydrolyzed substrate, as recommended by the manufacturer.

### 3.6. Osmotic and Oxidative Stress Tolerance and Cell Wall Stability of Candida

The test suspensions of *C. albicans* ATCC 10231 prepared from yeasts cultured on (a) SDA containing SAPFs at a final subMIC or (b) control SDA, were used. For osmotic stress tolerance testing, the volume of 5 μL of suspensions (at the densities of 10^5^, 10^4^, 10^3^ cells/mL) were spotted on SDA plates containing NaCl at the final concentrations of 1.0, 0.5 and 0.25 M. For the evaluation of the cell wall damaging agents activity, test inocula were spotted on SDA plates containing Calcofluor White (30.0–100.0 µg/mL). Macromorphology of *Candida* spot-growth (diameter and number of microcolonies) was monitored for 48 h, 30 °C and compared to the yeast growth on control plates, as described [29]. To test the oxidative stress tolerance, the *Candida* cell suspensions were treated for 1 h at 35 °C with hydrogen peroxide at the concentrations of 10, 25 or 50 mM. The next steps of the assay were identical as described above.

In order to test the effect of saponins on the cell membrane permeability, control and SAPFs—treated *C. albicans* cells (at concentration of 0.125, 0.25 mg/mL, up to 2 h) suspended in purified water (Sigma) were stained with propidium iodide (Molecular Probes, Eugene, OR, USA) at the final concentration of 60 µM, at room temperature. After 5 min at the dark, the residual dye was removed by centrifugation (1200 rpm) and samples were washed three times in PBS. The images of stained yeast were captured using a Confocal Laser Scanning Microscope (LSM510 META, Zeiss, Jena, Germany) combined with an Axiovert 200 M (Zeiss, Germany) inverted microscope equipped with a Plan-Neofluar objective (63x/1.25 Oil). All settings were held constant during the course of all experiments. The PI fluorescence was detected with a He-Ne laser (543 nm) and an LP filter set (560–615 nm) and images of the yeast were collected at Nomarski DIC at the excitation of 543 nm. Images from one representative experiment from two performed are shown in Figure 2.

### 3.7. C. albicans Germ Tube Formation and Spider Agar-Invasive Hyphal Growth under the Influence of SAPFs

To determine serum-induced germination and mycelium-like growth of *C. albicans* ATCC 10231, fresh suspensions of blastoconidia were incubated in RPMI-1640 containing 10% (v/v) of fetal calf serum, with or without the addition of subMIC of SAPFs. After the following time points: 1, 2, 3, 24 and 48 h, the percentages of germ tubes (GFT, hyphae or other forms of yeast cell morphology were evaluated. GFT percentage was calculated for 500 cells counted in several microscopic fields, after 1–3 h of incubation. After further incubation (24–48 h) morphology of yeast/hypha was microscopically assessed. Finally, the Spider test medium modified by the addition of SAPFs at subMIC (agar dilution) was used for the evaluation of invasive growth (medium without SAPFs served as a control). Macromorphology of mycelium formation was monitored daily for to 7 days of incubation at 30 °C, as described [30].

### 3.8. Determination of Anti-Mycotic Chemotherapeutics Synergy with SAPFs

The inoculum of *C. albicans* ATCC 10231 was spread on (a) SDA containing SAPFs at the final subMIC or (b) control SDA. The standard disk-diffusion test was performed according to the CLSI recommendations (document M44-A2), using the following anti-mycotics: amphothericin B (20 μg/disc), miconazole (10 μg/disc), clotrimazole (10 μg/disc), ketoconazole (10 μg/disc), nystatin (100 units), natamycin (10 μg/disc), econazole (10 μg/disc), flucytosin (1 μg/disc) (Mastring-S, Mast Diagnostics, Bootle Merseyside, UK). Growth inhibition zones were measured (HiAntibiotic Zone Scale, Emapol, Poland) after plates incubation at 37 °C for 24–48 h. Antibiotic gradient strips (E-test, BioMerieux, Marcy-l’Etoile, France) containing the representative anti-mycotic triazole—fluconazole were then used (FLC, the concentration range of 0.016–256 mg/L), according to the test manufacturer (on RPMI-1640 medium supplemented with 2% of glucose). Growth inhibition ellipsoid zones were measured (end-points were determined according to the manufacturer’s instructions). In this part of the study, besides *C. albicans* ATCC 10231 (MIC of 0.25 mg/L), *C. glabrata* clinical strain resistant to fluconazole (MIC of 64 mg/L) was used.

### 3.9. Statistical Analysis

Most of the values are expressed as means ± SD. Number of repetitions individual test was different and was given in the description of each of them, or in the results presentation. When applicable, statistical differences were evaluated using STATISTICA 6.0, USA. *p* < 0.05 was considered significant.

## 4. Conclusions

We believe that the main goal of our research, to demonstrate the possibility of reducing the virulence of *Candida* by subMIC of saponins has been achieved. The results showed that saponins isolated from aerial part of selected clovers have the desirable and safe potential being not hemolytic and cytotoxic, even when used at the concentration of 0.25 mg/mL, which was a potent inhibitor of *C. albicans* germ tubes formation, enzymatic activity and their invasive hyphal growth. It gives hope for the possibility of future use of the products as novel therapeutics supporting classic drugs in the course of fungal infections, formulated e.g., as ointment, lotion, a dressing or disinfectant [5,6,7,8,29,30,31,32,33,34].
